# Differences in risky sexual behaviors and HIV prevalence between men who have sex with men and transgender women in the Midwest Brazil

**DOI:** 10.1371/journal.pgph.0003061

**Published:** 2024-05-06

**Authors:** Gabriela Alves Cesar, Bárbara Vieira do Lago, Tayana Serpa Ortiz Tanaka, Priscila Brunini Zanini, Larissa Melo Bandeira, Marco Antonio Moreira Puga, Fernanda Rodas Pires Fernandes, Clarice Souza Pinto, Lisie Souza Castro, Lívia Garcia Bertolacci-Rocha, Carlos Eurico dos Santos Fernandes, Grazielli Rocha de Rezende, Ana Rita Coimbra Motta-Castro

**Affiliations:** 1 Laboratory of Clinical Immunology, Federal University of Mato Grosso do Sul, Campo Grande, MS, Brazil; 2 Laboratory of Viral Hepatitis, Oswaldo Cruz Foundation, Rio de Janeiro, Rio de Janeiro, RJ, Brazil; 3 Institute of Immunobiological Technology (Bio-Manguinhos), Fiocruz, Rio de Janeiro, Brazil; 4 Secretary of Health of the State of Mato Grosso do Sul, Campo Grande, MS, Brazil; 5 Federal University of Rondonópolis, Rondonópolis, MT, Brazil; 6 Faculty of Pharmacy, Federal University of Goiás, Goiânia, GO, Brazil; 7 Laboratory of General Pathology, Federal University of Mato Grosso do Sul, Campo Grande, MS, Brazil; 8 Laboratory of Molecular Virology, Oswaldo Cruz Foundation, Mato Grosso do Sul, Campo Grande, MS, Brazil; PLOS: Public Library of Science, UNITED STATES

## Abstract

Men who have sex with men (MSM) and transgender women (TW) are disproportionally affected by HIV infection. This cross-sectional study evaluated the HIV-1/2 prevalence, risk factors and HIV molecular features of MSM and TW from Midwest Brazil. Four hundred and thirty participants (278 MSM and 152 TW) from Mato Grosso do Sul, Brazil, were interviewed and tested for HIV-1/2 infection between November 2011 and September 2013. Participants who were assigned male at birth, older than 18 years old and self-declared as MSM or TW were recruited from LGBT+ associations, as well as public (parks, square, streets, etc) and private [nightclubs, saunas, brothels, etc] places. The prevalence of HIV-1 was 14.4% (9.0% among MSM and 24% among TW; p<0.001). The factor independently associated with HIV-1 infection among MSM was being 30 years-old or older. Among TW, having suffered sexual coercion, lifetime syphilis infection and hepatitis C virus exposure were associated with HIV-1 infection. Phylogenetic analyses classified 65% sequences as subtype B and 35% as possible recombinants. All but one recombinant sample were from TW individuals. High HIV-1 prevalences were observed in both groups, highlighting the urgent need to devise specific HIV interventions targeting these key populations. Notably, TWs are more vulnerable to HIV infection, which was associated with sexual violence and co-infection with other STIs. With regard to MSM, being 30 years old or older was significanty associated to HIV, reinforcing the idea that MSM are less exposed [or exposed later] to STIs than TWs, although MSM are clearly more vulnerable than the general population.

## Introduction

Since the first case of Acquired Immunodeficiency Syndrome (AIDS) in the world, in 1981, human immunodeficiency virus (HIV) infection has reached pandemic proportions. According to UNAIDS, it is estimated that 39 million (33.1–45.7 million) people are living with HIV/AIDS (PLWHA) worldwide. The median HIV prevalence among the adult population is around 0.7%. However, prevalence rates in key populations are much higher, reaching 7.5% in men who have sex with men (MSM) and 10.3% in transgender women (TW) [1].

In Brazil, the number of PLWHA untis estimated in 990,000 [2]. Although Brazil has made progress towards HIV control, achieving 88% of PLWHA diagnosed, 83% on antiretroviral therapy and 95% of these with suppressed viral load, inequalities are a limiting factor in reaching the UNAIDS 95-95-95 target. These inequalities prevent vulnerable individuals from having full access to HIV prevention and treatment, thus hindering the achievement of this goal [3].

Studies conducted in Brazil in different periods have shown a higher prevalence of HIV-1 infection among MSM and TW when compared to the general population [4–7]. A study carried out between 2011 and 2012 revealed an HIV prevalence of 15.4% in MSM living in São Paulo [5]. In agreement, a respondent-driven sampling (RDS) study conducted by Kerr et al. in 2009 in 10 Brazilian cities, found an overall HIV prevalence of 14.2% [6]. Similar data was also observed in a survey carried out in 12 Brazilian cities in 2016, revealing 17.5% overall HIV positivity [7].

The vulnerability of MSM and TW to HIV infection is likely to be multifactorial. Biologically, receptive anal sex can increase the risk of HIV infection due to the possibility of microlesions on the anal mucosa. In addition, vulnerabilities, such as discrimination, engagement in sex work and unprotected sexual practices have been linked to HIV infection in these populations [2,8–11].

Although previous studies have addressed HIV epidemiology among key populations in Central Brazil [6,7,12], the historical series of local data remains unknown. Investigating the prevalence and risk factors associated with HIV in MSM and TW a decade ago is crucial to understanding whether the interventions adopted in the past have led to a positive impact in the present. This cross-sectional study describes HIV prevalence in MSM and TW, highlighting the differences in sociodemographic characteristics, sexual behaviours and HIV molecular features between these key populations, a decade ago.

## Methods

### Study sample

This cross-sectional study was conducted in Campo Grande, Mato Grosso do Sul [MS], Central Brazil, between November 2011 and September 2013. This study was approved by the Research Ethics Committee involving Human Beings of Federal University of Mato Grosso do Sul (n° 158.931).

All the methodology regarding the recruitment of the participants was described in the study conducted by Fernandes et al. [13]. Briefly, the convenience sampling method was carried out to recruit two groups of participants, MSM and TW, through the self-designation during the interview at public (e.g., gay pride parades, square, parks, streets, etc.) and private places (e.g., bars, massage parlors, saunas, nightclubs, brothels, etc.) of the city. MSM and TW were previously contacted through the Mato Grosso do Sul State Association of Transvestites and Transsexuals and Reference Center for Human Rights in the Prevention and Combat of Homophobia. Informed consent about the study was signed from all participants. TW were defined as individuals who were assigned male at birth but had the opposite gender identity. Individuals who were assigned male at birth, older than 18 years old who self-reported sexual intercourse with other men in the last 12 months were eligible to participate.

Written informed consent was obtained from all participants. Posteriorly, participant’s data were collected through a face-to-face interview by trained professionals using a structured questionnaire regarding sociodemographic characteristics, such as alcohol and drug consumption (injection and non-injection drugs), history of blood transfusion, tattoos/piercings, and sexual behavior including age at first sexual intercourse, history of exchange sex for money, number of sexual partners in the last week, use of condoms with a male sexual partner, had experienced sexual coercion, performing different sexual practices (the practice of fingering/fisting, rimming (oral-anal *sex)*, sadism and/or masochism, shared sex toys, group sex, and others).

For all volunteers were offered free lubricant gel, condom packs, and risk reduction counseling about STIs. All participants who tested positive for HIV were referred to medical care at the Reference Center for Infectious and Parasitic Diseases from Campo Grande, MS.

### Serological tests

Afterward, blood specimens were obtained from each participant and tested for HIV, hepatitis B virus (HBV), hepatitis C virus (HCV), and syphilis infections. The HIV infection was determined using enzyme linked-immunosorbent assay (ELISA) (Murex, Kyalami, South Africa). EIA-reactive samples were confirmed by Western Blot assay (Novopath HIV-1, Immunoblot, Bio-Rad, Barcelona, Spain). Syphilis exposure was tested by using a treponemal antibody ELISA (ICE Syphilis1, DiaSorin, Italy). The HCV antibodies (anti-HCV) were investigated using ELISA–Murex Diagnostics, UK and confirmed by “line imunoassay” (INNO-LIA III HCV Ab, Innogenetics, Belgic). All serum samples were tested for the presence of HBV serological markers (HBsAg, anti-HBs and total anti-HBc).

### Statistical analysis

Information regarding sociodemographic characteristics and sexual behavior were recorded, double-checked, and entered into EPI-INFO 5.0 (Center for Diseases Control and Prevention, Atlanta, Georgia, USA, 1997) statistical software package. Data set of independent variables were previously categorized in subsets and crossbred analysis was prepared with the dependent variable (HIV positive or negative sample). The Stata 13.0 version software was used for data analysis. Samples were analyzed with the chi-square test to assess the difference in risk factors between MSM and TW. Univariate analysis was used to assess the association between independent and dependent variables. Variables with p <0.20 were included in the multivariate analysis. The association of exposure variables with HIV infection was explored using odds ratio in a multivariable logistic regression model. The analysis was stratified by gender identity (MSM and TW).

### Molecular and Phylogenetic analysis

For molecular analysis, cDNA from each sample was obtained from 200 μL of whole blood using the QIAamp DNA Blood Mini kit (Qiagen, Hilden, Germany) according to the manufacturer’s instructions. The partial polymerase gene including protease/reverse transcriptase (PR/RT) was amplified by nested polymerase chain reaction (PCR) and sequenced using combinations of primers previously described [14], resulting in a fragment of 1261 base pairs. Multiple sequence analyses were performed by using reference sequences from Los Alamos HIV Sequence Database1 employing the Clustal W program implemented in MEGA 7.0 software [15]. Recombinant profiles were inferred by bootscan analyses with a sliding window of 300 bp, steps of 10 bp and Kimura-2 parameters model using SimPlot 3.5.1 software. Phylogenetic, transmission clusters and drug resistence analyses were detailed in the study conducted by Tanaka et al. [16].

## Results

**Fig 1:**
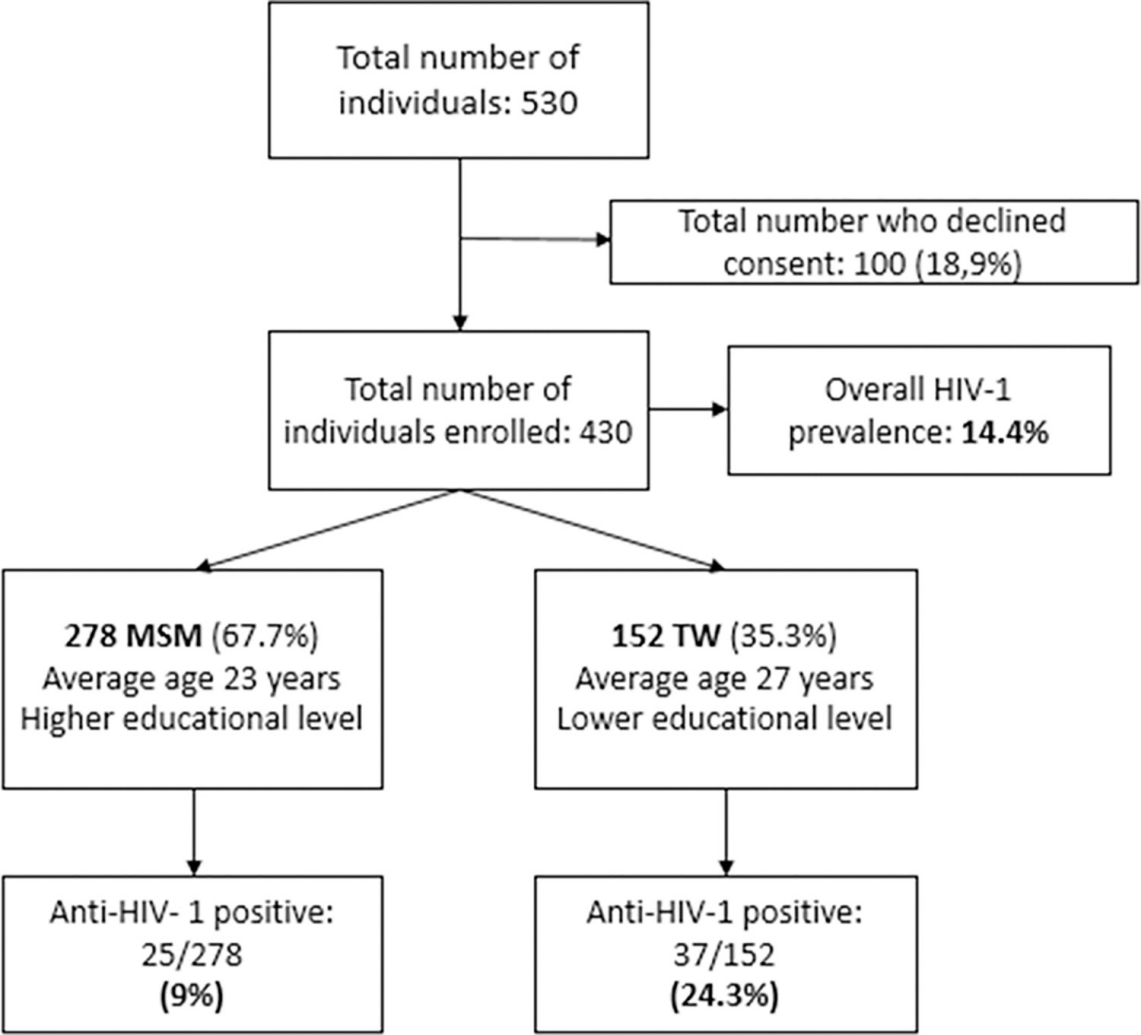
Flow chart of total number of subjects [MSM and TW] invited to the study.

Out of 530 individuals who were invited to participate in the study, 430 (81.1%) agreed to answer the questionnaire and collected a blood sample. Among the remaining 100 individuals who could not be included in the study, 30% were under use of alcohol, 20% were less than 18 years old, 10% did not have sexual intercourse with a man in the last 12 months and 40% did not want to be submitted to blood collection. Of the 430 eligible individuals, 278 (64.7%) were MSM and 152 (35.3%) were TW (Fig 1).

Marked differences between MSM and TW were observed for almost all variables, highlighting the importance of analyzing these participants separately.

Socio-economic, demographic, and behavioral characteristics of the two groups were previously described by Fernandes, et al, 2015. MSM average age was 23 years (from 18 to 61 years), while TW average age was 27 years (from 18 to 70 years). MSM and TW were predominantly unmarried. The level of education was found to be lower among TW (50.0% had less than 10 years of education) than among MSM (49.6% had 10 to 12 years of education). Overall, recreational drug use was reported by 63.8% of TW, while most MSM does not (59.4%). In addition, 52.5% of MSM had their first sexual experience before 15 years old, compared with 30.9% of TW. A total of 76.3% of MSM had one or no male sexual partner in the last 7 days, while about a half (46.7%) of TW reported more than 10 male sexual partners in the last 7 days. Both groups reported high frequency of irregular condom use in the last oral and anal intercourse. Had already received money or goods for sex was almost five times more frequent among TW (75.7%) than among MSM (18,8%). Both groups reported having sex with a female partner in the past 12 months and having ever experienced sexual coercion. Ever having engaged in different sexual practices was two times more frequent among TW (57.7%) than among MSM (27.5%) (Tables 1 and 2).

**Table 1:** Univariated and multivariated analysis of risk factors related to HIV infection in transgender women (TW).

TW (N = 152)						
Variable	HIV Pos/total	%	Odds ratio [IC 95%]	P	OD adjusted [IC 95%]	P
**Age group (years)**						
<20	2/24	8.3	Reference			
20–24	7/45	15.6	2.0 [0.4–10.6]	0.403	1.9 [0.3–14.0]	0.519
25–29	11/30	36.7	6.3 [1.2–32.4]	0.026	4.8 [0.7–36.2]	0.125
≥30	17/53	32.1	5.2 [1.1–24.7]	0.038	2.8 [0.4–21.2]	0.396
**Monthly household income (US$)** *						
>1448	1/13	7.7	Reference			
290–1448	18/72	25.0	4.0 [0.5–32. 9]	0.198	4.1 [0.4–39.3]	0.223
≤290	14/55	25.5	4.1 [0.5–34.4]	0.194	4.9 [0.5–48.9]	0.179
**Alcohol consumption in the last month**						
No	9/41	22.0	Reference			
Yes	28/111	25.2	1.2 [0.5–2.8]	0.676		
**Illicit drug use (ever)** *						
No	13/55	23.6	Reference			
Yes	24/97	24.7	1.1 [0.5–2.3]	0.879		
**Ever injected drugs**						
No	36/150	24.0	Reference			
Yes	1/2	50.0	3.2 [0.2–51.9]	0.419		
**Number of MSP in the last week**						
≤1	7/26	26.9	Reference			
2–10	7/31	22.6	0.8 [0.2–2.7]	0.705		
>10	17/69	24.6	0.9 [0.3–2.5]	0.819		
**Condom use with MSP in the last oral intercourse** *						
Yes	20/80	25.0	Reference			
No	6/26	23.1	0.9 [0.3–2.5]	0.843		
**Condom use with MSP in the last anal intercourse** *						
Yes	7/27	25.9	Reference			
No	19/79	24.1	0.9 [0.4–1.9]	0.845		
**Sex exchange for money and goods [ever]**						
No	7/37	18.9	Reference			
Yes	30/115	26.1	1.5 [0.6–3.8]	0.375		
**Had sex with FP in the past 12 months** *						
No	26/112	23.2	Reference			
Yes	7/30	23.3	1.0 [0.4–2.6]	0.989		
**Experienced sexual coercion (ever)**						
No	19/107	17.8	Reference			
Yes	15/35	42.9	3.5 [1.5–8.0]	0.003	5.1 [1.7–14.9]	0.003
**Sexual practices (ever)** * **/** **						
No	13/60	21.7	Reference			
Yes	20/82	24.4	1.2 [0.5–2.6]	0.704		
**Syphilis status**						
Negative	12/76	15.8	Reference			
Positive	25/76	32.9	2.6 [1.2–5.7]	0.014	3.0 [1.0–8.9]	0.046
**Hepatitis B (Anti-HBc)**						
Negative	17/110	15.5	Reference			
Positive	20/42	47.6	5.0 [2.2–11.0]	<0.001	3.1 [1.0–9.6]	0.052
**Hepatitis B (HBsAg)**						
Negative	35/148	23.6	Reference			
Positive	2/4	50.0	3.2 [0.4–23.8]	0.249		
**Hepatitis C**						
Negative	33/147	22.4	Reference			
Positive	4/5	80.0	13.8 [1.5–127.9]	0.021	15.7 [1.2–207.9]	0.037

*Missing

** Refers to rimming group sex and others

OD: Odds ratio. MSP: Male sexual partner, FP: Female partner.

**Table 2:** Univariate and multivariate analysis of risk factors related to HIV infection in men who have sex with men (MSM).

MSM [N = 278]						
Variable	HIV Pos/total	%	Odds ratio [IC 95%]	P	OD adjusted [IC 95%]	*P*
**Age group (years)**						
<20	2/70	2.9	Reference			
20–24	8/99	8.1	3.0 [0.6–14.5]	0.175	2.5[0.5–13.3]	0.263
25–29	5/49	10.2	3.9 [0.7–29.5]	0.176	3.7 [0.6–20.7]	0.140
≥30	10.6	16.7	6.8 [1.4–32.4]	0.016	5.6 [1.1–29.5]	0.043
**Monthly household income (US$)** *						
>1448	10/66	15.2	Reference			
290–1448	12/158	7.6	0.5 [0.2–1.1]	0.089	0.3 [0.1–0.9]	0.031
≤290	2/38	5.3	0.3 [0.1–1.5]	0.146	0.3 [0.1–1.5]	0.137
**Alcohol consumption in the last month**						
No	2/38	5.3	Reference			
Yes	23/240	9.6	1.9 [0.4–8.4]	0.548		
**Recreation drug use (ever)**						
No	13/165	7.9	Reference			
Yes	12/113	10.6	1.4 [0.6–3.2]	0.433		
**Number of MSP in the last week**						
≤1	12/118	10.2	Reference			
2–10	7/55	12.7	1.3 [0.5–3.5]	0.617		
>10	1/11	9.1	0.9 [0.1–7.5]	0.910		
**Condom use with MSP in the last oral intercourse** *						
´´Yes	2/21	9.5	Reference			
No	3/15	20.0	2.4 [0.3–16.4]	0.380		
**Condom use with MSP in the last anal intercourse**						
Yes	2/9	22.2	Reference			
No	3/29	10.3	0.4 [0.1–2.9]	0.368		
**Sex exchange for money and goods (ever)**						
No	20/234	8.5	Reference			
Yes	5/44	11.4	1.4 [0.5–3.9]	0.549		
**Had sex with FP in the past 12 months** *						
No	12/160	7.5	Reference			
Yes	10/95	10.5	1.5 [0.6–3.5]	0.405		
**Experienced sexual coercion (ever)** *						
No	18/224	8.0	Reference			
Yes	5/35	14.3	1.9 [0.7–5.5]	0.227		
**Sexual practices (ever)** *						
No	13/187	7.0	Reference			
Yes	9/71	12.7	1.9 [0.8–4.8]	0.141	1.5 [0.6–4.1]	0.370
**Syphilis**						
Negative	15/205	7.3	Reference			
Positive	10/73	13.7	2.0 [0.9–4.7]	0.102	1.8 [0.7–4.8]	0.258
**Hepatitis B (Anti-HBc)**						
Negative	21/249	8.4	Reference			
Positive	4/29	13.8	1.7 [0.6–5.5]	0.345		

*Missing

** Refers to rimming, group sex and others

OD: Odds ratio. MSP: Male sexual partner, FP: Female partner.

The overall HIV-1 prevalence was 14.4% (62/430; 95% CI: 11.3 to 18.2). However, HIV-1 prevalence was significantly higher in TW (24%; 37/152; 95% CI: 17.5 to 31.2) than in MSM (9.0%; 25/278; 95% CI: 5.6 to 12.4) (*p*<0.001).

Univariate and multivariate analysis were conducted in order to access the risk factors associated with HIV-1 infection. Among TW, have been experienced sexual coercion, lifetime syphilis infection and HCV exposure were associated with HIV-1 infection (Table 1). Among MSM, after adjusting for significant variables identified on univariate analysis, be 30 years of age or older was independently correlated with HIV-1 infection (Table 2).

From all 62 HIV positive individuals, 51.6% (32/62) tested positive for cDNA by qualitative PCR and 20 had detectable HIV-RNA in the quantitative test. Mean viral load was 5.57 ± 1.42 log copies/mL. All twenty HIV-RNA positive samples were sequenced, 9 from MSM and 11 from TW. Phylogenetic analyses classified 65% (13/20) sequences as subtype B and 35% (7/20) as possible recombinants (URF_BF1: 2, BF1:1, CF1:1, BD:1, BC:1 and F1C:1). All but one recombinant sample were from TW individuals. In addition, all recombinant samples were found in individuals who presented at least one sex-related risk factor and/or tested positive for a sexually transmitted infection other than HIV. Molecular characteristics, co-infections and risk factors of sequenced individuals are presented in Table 3.

**Table 3:** Molecular characteristics of HIV sequenced samples.

Sample	Age range	Self-designation	Viral load (log copies/mL)	CD4	HIV-1 Subtype	Co-infections	Risk factors
**H851**	20–29	TW	3.817	674	B	-	SW, DSP
**H853**	20–29	TW	ND	ND	F1/C	past HAV, past HBV	SW, ICU, transfusion
**H855**	20–29	MSM	4.684	629	B	-	-
**H859**	20–29	MSM	3.676	13	B	-	-
**H860**	20–29	MSM	4.289	1.188	B	Lifetime syphilis	SW, ICU, DSP
**H861**	20–29	TW	4.185	333	B	Lifetime syphilis	SW, ICU, DSP
**H862**	20–29	MSM	3.990	459	B	-	-
**H867**	30–39	MSM	NI	NI	BD	past HAV,	ICU, DSP
**H871**	30–39	TW	4.228	593	BF1	past HAV, past HBV, HCV	SW, ICU, IDU, DSP
**H872**	70–89	TW	UND	683	B	Lifetime syphilis	ICU
**H873**	30–39	MSM	4.713	547	B	Lifetime syphilis	Transfusion
**H874**	20–29	MSM	NI	NI	B	Lifetime syphilis	ICU
**H875**	20–29	MSM	4.542	308	B	past HBV	ICU
**H876**	20–29	MSM	5.053	217	B	Lifetime syphilis, past HBV	SW, ICU, DSP.
**H877**	20–29	TW	ND	ND	BF1	past HAV, lifetime syphilis, past HBV	SW, ICU, DSP
**H878**	20–29	TW	5.742	204	B	Lifetime syphilis	DSP
**H879**	20–29	TW	1.987	365	BF1	past HAV, lifetime syphilis	SW, ICU, transfusion,
**H880**	≤19	TW	3.819	639	CF1	past HAV	DSP
**H881**	20–29	TW	5.451	512	B	past HBV	SW, ICU,
**H882**	20–29	TW	3.911	389	BC	past HAV, lifetime syphilis	SW, ICU,

ND: Not done [no sample available]; SW: Sex worker; IDU: Injectable drug user; ICU: Irregular condom user; DSP: Different sexual practice.

## Discussion

Although there is reliable epidemiological information on HIV infection within the LGBT+ community in Brazil [6,7,12,17,18], this is the first study conducted in the Central Brazil, addressing HIV prevalence, risk factors and molecular epidemiology of MSM and TW as separate populations, considering their distinct realities.

According to information provided by UNAIDS, about 0.43% of the Brazilian population are living with HIV [2]. Our study reveals that the overall HIV-1 prevalence in MSM and TW (14.0%; 95% CI: 11.3 to 18.2) was almost thirty-three times higher than that found in the general population at the same time, confirming that the HIV epidemic in Brazil is disproportionately concentrated among these key populations. Although this study was conducted a decade ago, data provided by others (including current official data) demonstrated that these alarming prevalences have not changed over time [4,7,12,19,20]. Despite being a cross-sectional study and not allowing comparisons with RDS studies, the high HIV-1 prevalence found in this study were also observed in studies conduted in these same groups in Salvador (6.3%) in 2008 [21], in 10 Brazilian cities (14.2%) in 2009 [6], in 12 Brazilian cities (17,5%) in 2016 [7], in MSM from Central Brazil (17.6%) in 2014 [12] and in TW from Northeast Brazil (9.0% and 24.3%) from 2014 to 2016 and 2016 to 2017, respectively. Similarly, when comparing our work to studies conducted in other countries with different methodologies, we also found similar data in Colombia [22] 15.0% (95% CI: 11.3 to 17.8) and lower prevalences in Rhode Island [23] 5.0% (95% CI: 2.1 to 8.7) and Ukraine [24] 7.8% (95% CI: 3.1 to 12.3). These findings together show no significant reduction in HIV prevalence over time. Therefore, it is reasonable to assume that interventions designed in the past may have failed to reach these key populations by not fully embracing their peculiarities.

Notably, HIV prevalence was much higher in TW than in MSM (24.0% vs. 9.0%, *p* <0.001) and was 55.8 times higher than in the general population. These discrepancies may reflect social determinants of health, differences in sexual risk behaviors and other vulnerabilities, including difficulties to access public healthcare services, gender identity-related stigma, and violence [25,26]. Assessing the sociodemographic profile of the two study groups (Tables 1 and 2), it is clear that social determinants such as lower education level, illicit drug use, higher number of sexual partners, engagement in sex work and other vulnerabilities observed in TW may play a role in the alarming HIV prevalence observed in this group. Studies conducted in Brazil have reported extremely high HIV prevalence in TW, ranging from 12–78% [17–20]. A meta-analysis of HIV prevalence from 15 countries demonstrated that the odds ratio of a TW being HIV positive is 48.8 when compared to other reproductive age adults [25]. In agreement, a cross-sectional study conducted in Brazil demonstrated that the odds of HIV infection in TW compared with the general Brazilian population was 55.5 [27]. In fact, many authors have reported that TW often face stigma and discrimination in the health care environment, leading many of them to avoid health services, including HIV/AIDS prevention programs [27,28]. It has been demonstrated that Brazilian TW are at high risk of multiple ways of violence [20,28]. According to Benevides & Nogueira (2019) [26], the estimated life expectancy of TW is 35 years old in contrast to 75 years-old for the general Brazilian population. These differences may be due to alarming murder rates of TW in Brazil and, in a lesser proportion, poor health determinants and suicide.

The prevalence and risk factors for HIV in TW reported in this study [24%; 95% CI: 17.5 to 31.2] was similar to findings from other countries [27,29–31]. In the present study, sexual violence was associated with HIV-1 infection in this group (p <0.003). History of sexual abuse, which usually occurs without condoms, leads to an increased risk of HIV infection and other STIs. In addition, this population is targeted for prejudice, family exclusion, unstable housing and unemployment, often leading TW to engage in sex work. The criminalization and stigma of sex work, a job reported by 75.7% of TW, have left them more vulnerable to physical, psychological and sexual violence [32–34]. Completing this picture, lifetime syphilis infection and exposure to hepatitis C virus were statistically associated with HIV-1 infection in TW. In these cases, similar modes of transmission, irregular condom use, sexual violence and, more directly, the lesions caused by syphilis may increase the exposure to HIV [13,35–37]. Taken together, these findings pointed to a extremely high vulnerability faced by TW which might explain the discrepancy in HIV prevalence. From a public health perspective, social and structural interventions such as improving access to formal education, expanding TW representation in key positions where they can design and manage public health policies can be the cornerstone for overcoming this alarming HIV rates and other health issues.

Regarding the MSM group, being aged 30 or over was independently correlated with HIV-1 infection. This data was explored by Dong et al. [38] in a large-scale systematic analysis. According to the authors, the high HIV prevalence in older MSM may be attributed to increased exposure to HIV over time and to higher rates of unprotected anal sex. Indeed, the existing high prevalence of HIV-1 infection among MSM, as reported by other studies, enhances the risk of being exposed to HIV in every sexual encounter [39,40].

In this study, we revisited the molecular data partially published by Tanaka et al. [16] who provided a phylogenetic detailed description of MSM population. Here, however, we focused on the epidemiological aspects of HIV-1 infected individuals and described their molecular features, highlighting the striking differences between MSM and TW.

Our results demonstrated that 65% of sequences were classified as HIV-1 subtype B, followed by 35% of recombinants. All subtype B sequences were classified as pandemic B. High frequency of recombinants have been described by other authors for different HIV-infected populations, including Brazilian MSM [12,41–44]. Here, all but one recombinant were detected in TW. All of them are sex workers, irregular condom users and presented at least one co-infection. These findings together suggested that, due to the increased risk behaviors and vulnerabilities to which this population is continually exposed [26,27,31,35–37], TW are at the nexus of the emergence of recombinant viruses. Despite the limited number of sequenced samples precludes deeper conclusions, it is reasonable to admit that the higher HIV-1 genetic complexity observed in TW may lead to consequences for public health, once the selection and spread of new recombinants may impact disease progression, viral persistence, and resistance to antiviral therapy.

This cross-sectional study has some limitations. This study was conducted 10 years ago, so the data presented here are not new, although they still reflect the current epidemiological scenario of HIV infection in these key populations. In addition, the convenience sampling and the study design cannot be used to infer population estimates or behavior over a period of time and may not provide definite information about cause-and-effect relationships. It was carried out in a limited number of TW and MSM from the capital of Mato Grosso do Sul State, Central Brazil, which does not represent Brazil as a whole. In addition, self-reporting and recall bias are also limitations of the present study. Moreover, our statistical findings did not allow us to clearly distinguish the vulnerabilities faced by MSM and TW. A possible explanation is that many participants failed to respond to some questions, causing missing in the database. As discussed above, violence in health services experienced in the past may lead members of these groups, especially TWs, to provide incomplete or biased information that prevents a statistically supported conclusion about the vulnerabilities faced by each of these populations separately.

Despite the relatively small sample size, which is not representative of the entire population, this study provides a historical perspective of HIV infection in MSM and TW in Central Brazil drawing attention to a hard-to-reach population, for which structural interventions focusing on embracing differences as a key health determinant must be effectively addressed.

## Conclusion

A high prevalence of HIV was observed in both MSM and TW when compared to general population. Differences in vulnerabilities faced by MSM and TW exist and may reflect the discrepancy in HIV-1 prevalence observed between them (24% in TW vs. 9.0% in MSM). The variables statistically significant to HIV infection in TW were being forced to sexual intercourse, HCV exposure, and active syphilis infection. Taken together, these findings suggest that TW are more vulnerable to sexual violence and are more exposed to STI than MSM, which might explain the discrepancy in HIV prevalence. For MSM group, the only statistically significant variable was being 30 years-old or older, which reinforces the idea that MSM are less exposed (or exposed later) to STIs such as HIV than TW. While these variables may not provide definitive conclusions about cause-and-effect relationships, our findings from a decade ago show that measures devised in the past may not have led to considerable improvements in the prevention and control of HIV infection today.

To achieve the UNAIDS 95-95-95 target for HIV control, multisectoral rights-based strategies involving different social segments must be adopted. Effective affirmative actions such as job promotion, gender affirmation policies, increased political and social representativeness, combined with harm reduction programs for drug users and HIV-direct-acting measures could effectively close gaps that have been open for well over a decade.
